# Reduced habit-driven errors in Parkinson’s Disease

**DOI:** 10.1038/s41598-019-39294-z

**Published:** 2019-03-04

**Authors:** Colin Bannard, Mariana Leriche, Oliver Bandmann, Christopher H. Brown, Elisa Ferracane, Álvaro Sánchez-Ferro, José Obeso, Peter Redgrave, Tom Stafford

**Affiliations:** 1 Department of Psychological Sciences, University of Liverpool, Liverpool, UK; 2 Department of Anatomy, University of Otago, Dunedin, New Zealand; 3 Sheffield Institute for Translational Neuroscience (SITraN), University of Sheffield, Sheffield, UK; 4 Department of Linguistics, University of Texas at Austin, Austin, USA; 5 HM Hospitales, Centre for Integrative Neuroscience AC, Hospital Universitario HM Puerta del Sur, Mostoles and CEU San Pablo University. Center for Networked Biomedical Research on Neurodegenerative Diseases, Institute Carlos III, Madrid, Spain; 6 Department of Psychology, University of Sheffield, Sheffield, UK

## Abstract

Parkinson’s Disease can be understood as a disorder of motor habits. A prediction of this theory is that early stage Parkinson’s patients will display fewer errors caused by interference from previously over-learned behaviours. We test this prediction in the domain of skilled typing, where actions are easy to record and errors easy to identify. We describe a method for categorizing errors as simple motor errors or habit-driven errors. We test Spanish and English participants with and without Parkinson’s, and show that indeed patients make fewer habit errors than healthy controls, and, further, that classification of error type increases the accuracy of discriminating between patients and healthy controls. As well as being a validation of a theory-led prediction, these results offer promise for automated, enhanced and early diagnosis of Parkinson’s Disease.

## Introduction

Parkinson’s Disease (PD) is a neurodegenerative disorder. The cardinal features, bradykinesia, tremor and rigidity, are driven by dysfunction of the basal ganglia, specifically the loss of dopaminergic inputs. Diagnosis is primarily based upon these motor symptoms, which do not appear until a significant loss of dopaminergic neurons from their source, the substantia nigra pars compacta, has already occurred^1^. Recent results have suggested a particular spatial pattern in the progression of loss of dopaminergic neurons — starting in the ventrolateral substantia nigra and their terminals in the caudal putamen^2,3^. Therefore, a test for the early loss of function, specifically in the caudal putamen, could serve as an early marker for PD.

The basal ganglia receives inputs from functionally segregated regions of cerebral cortex in a topographically-organised manner^4^. Therefore, cortical regions associated with limbic (i.e. motivational and emotional), associative (i.e. cognitive) and sensorimotor functions access spatially ordered territories extending from rostro-ventromedial to caudo-dorsolateral zones of the striatum. Current evidence^5,6^, indicates that goal-directed and habitual control of behavior can be mapped respectively onto the associative (caudate and rostral putamen) and sensorimotor (caudal putamen) territories of the striatum. Under voluntary goal-directed control, actions are selected on the basis of relative outcome values, while automatic stimulus-response habits are selected according to relative stimulus salience, independent of outcome value^7^.

Given the early degeneration of ventrolateral dopaminergic cells, and given that these cells project to areas which support habitual action, our proposal in 2010 was that Parkinson’s disease would be expected to prevent patients from engaging their automatic everyday habits^5^. Thus, with a progressive loss of rapid low-cost habitual action control, patients would have to rely increasingly on their slower, effortful voluntary goal-directed control system. Recent experimental work and the clinical literature contain much evidence supporting this notion^8–10^.

This now well-supported description of malfunction within the basal ganglia^5^ provides the theoretical basis for the experiment reported here. The idea is to develop a behavioural assay that provides a quantitative index of the functional status of the caudolateral putamen^2^. The challenge is to come up with an easy and simple test that would give early warning that habitual control is starting to degrade.

Our solution is to exploit the phenomenon of ‘action slips’^11^. These are mistakes where we do something out of habit, but at an inappropriate time. An example would be, while deep in thought, taking the lift to your old office rather that to the floor where your new office is located. Such mistakes satisfy the independence of outcome-valuation criterion for habits^7^. A paradigm where we think action slips can be brought under tractable experimental control are the mistakes we all make while typing on computers, tablets and smart-phones.

Stimulus-response habits develop when predictable sequences of behaviour are repeated^7^. Thus, any frequently-used sequence of key-strokes (such as i-n-g) will gradually become habitual. Obviously practice typing will train the motor system to absorb a host of statistical regularities associated with reappearing patterns in the language typed. Our project is based on the prediction that habitual control of frequent key-stroke patterns by PD patients should start to disappear as the dopamine loss progressively impairs the automatic sensorimotor function of the caudal putamen. This means that alongside less fluent typing overall, PD should be marked by a decrease in the habit driven errors (such as typing t-h-i-n-g when the intended word is t-h-i-n-k). This prediction is marked in that it specifies decreases in one particular type of error committed by patients. Obviously to predict an increase in error, even an error of a specific type, would be less interesting for a disease predominantly characterised by dysfunction of the motor system. However while our theory leads us to predict a general increase in motor errors, the present investigation tests whether there is a specific decrease in habit-driven errors in the typing of participants with Parkinson’s Disease.

As well as testing this theory driven prediction, our experiment aims to lay the foundation for continuous, unobtrusive, quantitative monitoring of people’s dependence on habitual control. The advantage of typing is that, increasingly for many people, it is an everyday behaviour that potentially affords the unobtrusive measurement of a high volume of movement related data. With the habit error test we hope to lay the foundation for an automated analysis to detect caudal putamen dysfunction as an indication of early Parkinson’s. If successful, we would have an objective patho-physiologically inspired test for the early detection of Parkinson’s disease. We may also have a procedure that provides insight into the mechanisms of action of current and future therapies for Parkinson’s.

## Results

By taking the opportunity to test at two sites — one in England and one in Spain — we are able to seek support for the language-independence of our methods. The results from both sites are in line with one another, but we present key measures separately.

First we look at interkey interval (IKI, a standard metric for assessing typing speed), for both correct key presses and errorful key presses. In order to exclude mid-sentence breaks from consideration, we start by excluding any keypresses that are greater than the 99.5th percentile for correct keypresses in each of the two languages — all interkey intervals greater than or equal to 2973 ms for English and 2476 ms for Spanish. For errorful key presses this results in the removal of 1.4% of the English data and 3.9% of the Spanish data. Of the removed errors 69% were from patients.

**Figure 1:**
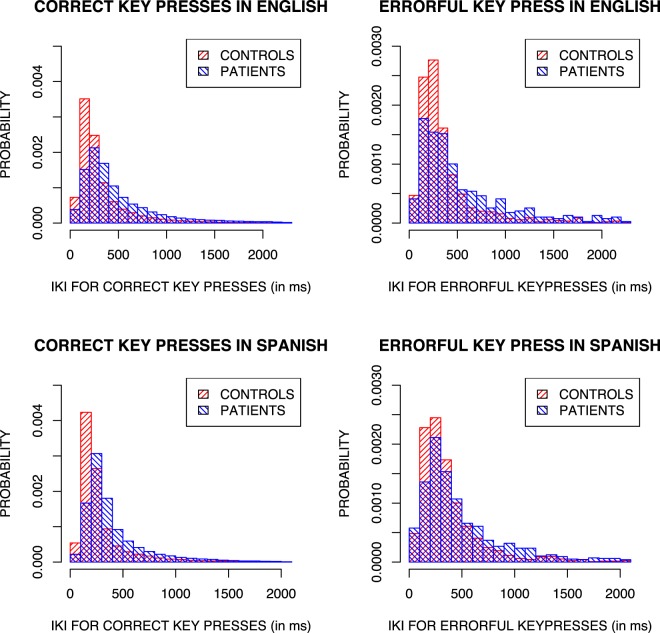
Distribution of interkey intervals for correct and errorful keypresses in English and Spanish patients and controls.

The timing of correct and errorful keypresses in both language and populations is shown in Fig. 1. As can be seen, patients take longer to perform both correct and errorful keypresses than non-patients for both languages. A 2 × 2 × 2 mixed ANOVA confirmed there to be an effect of keypress type (Correct or Errorful; F(1,57) = 39.67, p < 0.001) and participant group (Patient or Control; F(1,57) = 20.19, p < 0.001) on a participant’s mean interkey interval, but no significant effect of language and no significant interactions.

Each error made was next classified as being a motor error, a habit slip or neither. An error was classified as a motor error if the key pressed in error was closer to the previous key than to the target (correct) key. An error was classified as a habit slip if it was not a motor error and the key pressed in error had a higher conditional probability given the previous keypresses than the target key. See our method section for further details. The proportion of a participant’s full set of errors that were motor errors was then taken as their motor error rate, and their proportion of non-motor errors that were habit slips were taken as their habit slip rate.

**Figure 2:**
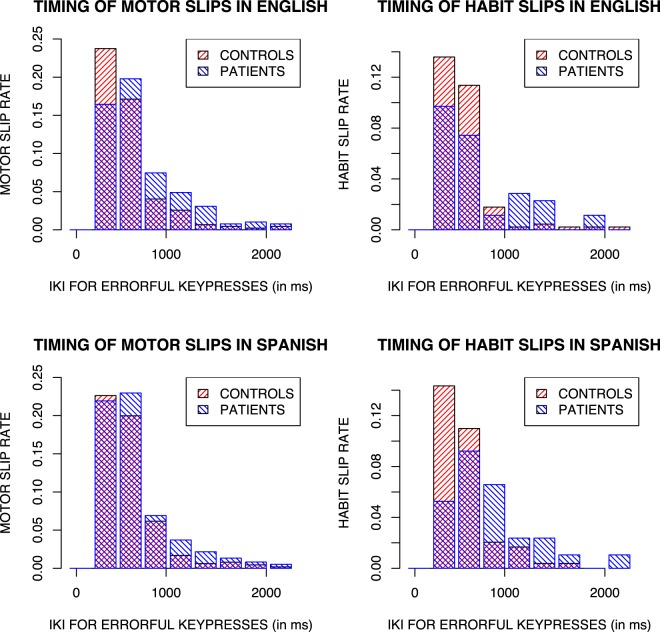
Distribution of motor and habit slip errors at different interkey intervals for patients and controls in English and Spanish. Note that habit errors rate is a proportion of errors that are not motor errors.

Our end goal is to look at the relationship between patient status and participants’ patterns of errors. However it is also important to understand the relationship between error type and timing. The proportion of each type of key press error for each interkey interval can be seen for each of the two languages in Fig. 2. We found above that participants’ mean interkey intervals were longer for errorful key presses than correct key presses but that this didn’t vary by patient status. Here we look at the relationship between timing and error type, specifically asking whether a participant’s mean IKI for errors differs depending on the rate with which they produce the two kinds of errors. Multiple regression modelling with model comparisons used to evaluate the significance of terms reveals that as well as the effect of patient status (F(1,56) = 12.06, *p* < 0.001) on mean IKIs for errors there was also an effect of habit slip rate (F(1,56) = 9.82, *p* < 0.01) but no effect of motor error rate or language.

**Figure 3:**
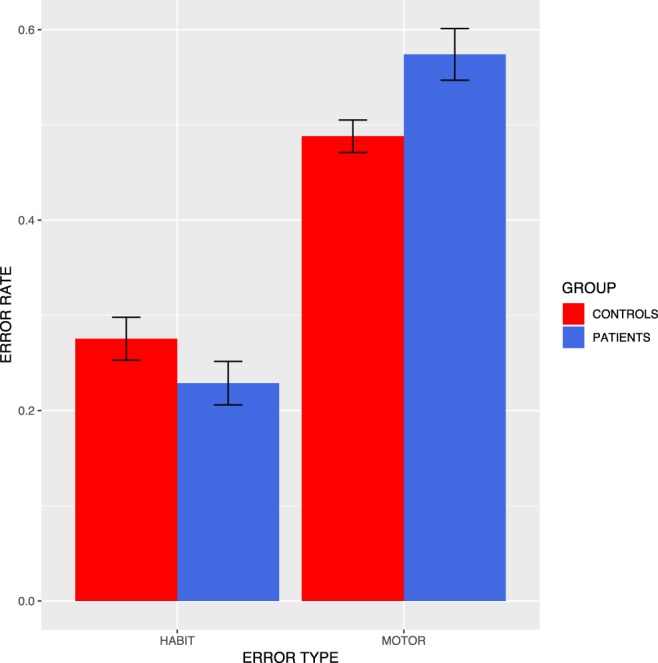
Rates of error types by participant group, collapsing over time points and language. Note that habit errors rate is a proportion of errors that are not motor errors.

A summary of the timing and error proportions can be seen in Table 1, and the relationship between patient status and error type collapsing over time and language can be seen in Fig. 3. The difference in habit error rate between patients and controls is significant (t(59) = 1.715, *p* < 0.05).

**Table 1:** Mean error rates and interkey intervals by language and participant group. Standard deviations are in parentheses. Note that habit errors rate is a proportion of errors that are not motor errors.

	Motor Errors		Habit Errors		Correct keypress IKI in seconds		Error keypress IKI in seconds	
	Controls	Patients	Controls	Patients	Controls	Patients	Controls	Patients
English	0.481 (0.108)	0.564 (0.142)	0.274 (0.146)	0.200 (0.141)	0.322 (0.120)	0.578 (0.267)	0.402 (0.148)	0.774 (0.474)
Spanish	0.522 (0.105)	0.588 (0.078)	0.320 (0.135)	0.269 (0.070)	0.288 (0.043)	0.435 (0.112)	0.389 (0.076)	0.545 (0.144)

We next look at whether the error rates observed can be used to predict the patient status of participants. We do this by first using a regression model to predict UPDRS scores and then looking at the utility of our predicted scores in distinguishing patients from controls. As there is no reason to assume that the predictors have a linear relationship with the UPDRS score we use generalized additive regression models, and as the UPDRS score is heavily right skewed we employ a poisson link. The mgcv package^12^ was used to fit these models with the default thin plate regression splines used to represent the smooth terms. A model including language plus each participants mean IKI for correct and for errorful key presses and both motor error and habit slip rates was found to give a better fit to the data that a model with any of these predictors removed. Fit was estimated using AIC, and we can infer from the differences between fits that the plausibility that the subset models give as good a fit as the full model is “essentially none”^13^. The full model explained 75.6% of the deviance.

**Figure 4:**
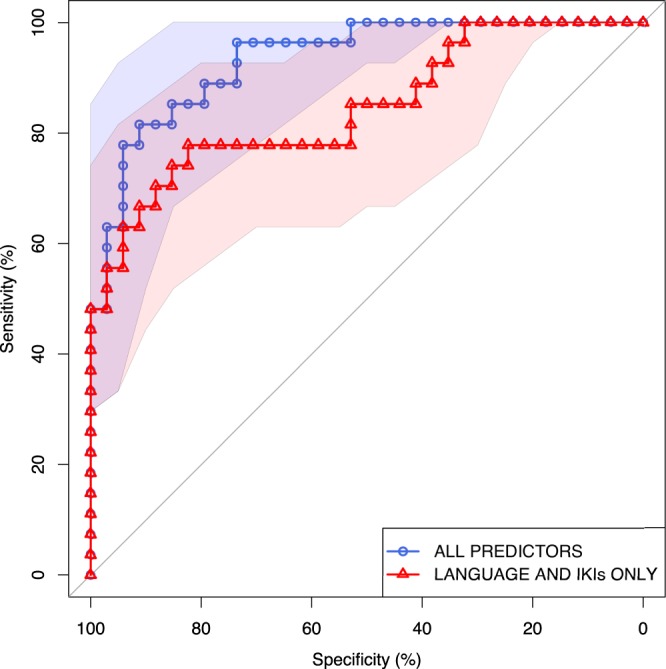
Receiver operating characteristic (ROC) curves for classification of participants as having Parkinson’s diagnosis or not. Two models shown with bootstrapped 95% confidence intervals: using all predictors, including rates for different error types (AUC = 0.954), and only using average speed of correct keypresses, average speed of errorful keypresses and language (AUC = 0.874). The AUC of the all predictor curve is significantly greater than that making use of the reduced predictor set (*p* < 0.05; DeLong’s test). A model including habit slip rate (and language and IKI predictors) but not motor slip rate also gives a significant improvement over the reduced model (AUC = 0.947; *p* < 0.05).

Finally we look at the value of the UPDRS scores predicted by our GAM models in distinguishing patients from non-patients. We do this by exploring a range of thresholds for diagnosis to produce the sensitivity vs specificity plot seen in Fig. 4. This shows the classification performance of our full model and a model with only language and IKIs included as predictors. The AUC of the all predictor curve (AUC = 0.954) is significantly greater than that making use of the reduced predictor set (AUC = 0.874; *p* < 0.05; DeLong’s test). A model including habit slip rate (and language and IKI predictors) but not motor slip rate also gives a significant improvement over the language-and-IKI-only model (AUC = 0.947; *p* < 0.05).

## Discussion

As predicted, patients with Parkinson’s commit fewer habit errors than healthy controls. This is in the context of the expected increase in simple motor errors and general slowing.

By using errors rates to perform classification we have demonstrated that typing data alone allows disease status to be identified. The model shows that both habit errors and typing speed contribute to this classification: accuracy is enhanced when habit slip rate is added to simpler models which use only speed information, or speed information and motor error rate, suggesting that the classification using habit slip rate adds additional information beyond that of motor speed alone.

While patients typed more slowly than controls in general, participants who typed more slowly were not less likely to make motor errors. However those who typed more slowly *were* less likely to make habit slips. This habit-specific speed-accuracy trade-off is additional evidence for a distinct control system for habits working within overall motor control of typing. It also raises the possibility that decreased habits in patients are simply the result of slower typing in patients - that they make fewer habit error simply because they type more slowly. While not inconsistent with our hypothesis, such an interpretation would complicate the causal relationship we have proposed. It is important to note then that habit slip rate was found to be of predictive value over and above timing information, suggesting that there is a clear separable relationship between habit slips and participant group.

These results provide compelling evidence supporting our hypothesis^5^. In both languages tested, English and Spanish, patients committed fewer habit errors than healthy controls but made more simple motor errors. This suggest that the habit system in PD patients is impaired, causing them to have to rely more on voluntary goal-directed control. Consistent results at both testing sites, and in two languages, suggest that the result is not language-specific and so has potential for further generalisation beyond Spanish and English.

This method has the potential for early diagnosis of PD for two reasons. First, because of results suggesting that the neural substrate of the habit system is first to be affected in the progression of Parkinson’s, a measure which is based on the loss of habit errors is well positioned to be sensitive to the first behavioural signs of the disease. Second, because typing is an everyday activity which can be unobtrusively and easily monitored, providing a very high volume of psychomotor behavioural data for any potential test.

While promising, the result reported suffers from some limitations as well. It was conducted with patients up to five years after they were diagnosed, with Parkinson’s Disease, rather than pre-, or at the point of, diagnosis. The sample size is limited and the result awaits replication by a preregistered trial. Furthermore, it remains to be seen if this method will generalise to non-laboratory conditions and to free text entry rather than copy-typing. Next steps to obtain further support for the hypothesis could include: larger studies with clinical populations and/or prospective studies with at risk populations, such as people with REM sleep behaviour disorder^14,15^.

Earlier diagnosis is one potential benefit of a typing test of Parkinson’s. Others are the role in confirmation of initial diagnosis (given high levels of misdiagnosis reported at some sites^16^) and as a measure of disease state or progression which is not reliant on subjective or time-consuming clinical assessment. A sensitive behavioural measure of disease state would accelerate the development of effective treatments.

Other authors have suggested typing tests for Parkinson’s disease^17–20^. Our task involves higher level cognitive function than Noyce *et al*.’s finger tapping task, and so has the potential to identify dysfunction in a richer array of cognitive systems. Giancardo *et al*.’s^19^ algorithm is less transparent as to what specific features of behaviour are affected in Parkinson’s and so contribute to the performance of the classification algorithm.

Cognitively and neurophysiologically informed behavioural testing holds great promise for both understanding the symptoms of Parkinson’s, and in contributing to diagnosis and management.

## Methods

### Ethical oversight

All experimental protocols were approved by NHS Health Research Authority (no. STH18662TK) and HM Hospitales, Spain (no. 14.11. 710-GHM). Informed consent was obtained from all subjects involved in the study. All the experiments and recruitment were carried out in accordance with the relevant institutional guidelines.

### Participants

Patients were recruited to be in the early stages of PD (Hoehn-Yahr stages 0–2.5, UPDRS <20 in the medicated state if not medication naive), with normal cognitive function (MMSE 27–30) and with less than 5 years from a confirmed diagnosis. Demographic information is shown in Table 2.

**Table 2:** Demographic and clinical information of study participants.

	N	Average age	N female	Education (years)	MMSE	UPDRS	Hoehn-Yahr
Controls	34	58.7	17	17.2	29.6	0.85	0
Patients	27	60.7	10	17.2	29.4	13.15	1.85

We excluded participants with cognitive impairment or dementia, participants unable to use a keyboard (e.g. due to upper limb functional limitation). Two control participants were excluded from the analysis because their UPDRS scores were in the same range as the patients.

Most patients (21 out of 27) were tested prior to taking their morning medication. These patients were retested in the same visit, 1.5–2 hours after after taking their medication (not reported here). Six patients were tested medicated (1 on a MAO-B inhibitor (rasagiline); 1 on a dopamine agonist (mirapexin); 4 on both (rasagiline and rotigotine, pramipexol or mirapexin).

### Procedure

All tests were completed in one visit. Evaluation of motor symptoms and cognitive state were measured by applying the UPDRS (Part III) and MMSE. The experiment was designed to run in a typical web browser, so we could easily move to testing online if required, and to demonstrate that this detection mechanism could be widely deployed, without requiring specialized hardware. Bespoke software was written which presents a series of sentences to be copy-typed, and collects identity and timing of each key-press. This software has been released as open-source at https://github.com/chbrown/typing-evaluation, which includes all the required code and instructions for running it, but omits the specific stimuli data we used. The outcome of each keypress appeared on the screen, as in normal computer typing (note that some experiments on typing restrict visual feedback^21^). If participants did not complete copy-typing the full set of sentences within 20 minutes they were invited to stop when they wished. In addition to the copy-typing measures, participants were also asked to participate in another experimental task which involve navigating an online road in the manner of a driving game (not reported here), and to complete some simple key- tapping exercises in order to collect the measures validated by^17^.

### Materials

For stimuli we sought naturally occurring sentences on which we knew people were likely to make typing errors. Our sentences were taken from a much larger set of sentences used to study typing errors (as part of a larger project) because they met this need. These sentences were identified using Wikipedia revision logs. Errors were extracted from pairs of sentences and their revisions, by assuming that older sentences contain errors and new versions of these sentences correct these errors. We performed this by applying the Wikipedia Revision Toolkit^22^ to the Wikipedia revision dumps downloaded from http://download.wikimedia.org/. Revision pairs that contained an edit to a single word token and where the replacement token was a real word were considered candidates for inclusion. A total of 15 sentences in English were chosen for the English participants. 30 sentences in Spanish were chosen for Spanish participants. Sentences ranged in length from 10 to 25 words.

For the identification of habit errors, character-based 5-gram language models were build using the SRILM toolkit^23^. The English and the Spanish n-gram models were estimated from the English and Spanish components of the Europarl corpus^24^. Back-off smoothing was used when the typed character n-grams did not occur in the corresponding corpus.

### Analysis

#### Error classification

An alignment between the typed sequence and the target sequence was found using the Needleman-Wunsch algorithm^25^ with scores of 1 for a match, -1 for a substitution and -2 for an insertion or deletion. The algorithm uses this scoring to maximise an alignment score (the inverse of an edit distance), and so return two full-sentence sequences of equal length with either matching items for corresponding positions in the sequences, mismatching items for corresponding positions (substitutions), a character in the typed sequence and a skip character in the target sequence (insertions), or a character in the target sequence and a skip character in the typed sequence (deletions). Sequences of typed characters were then paired with target words, by moving backwards from the end of the full typed sequence, concatenating characters to form tokens, and inserting token boundaries where there was a space character in the target sequence that was paired with anything but a skip character in the typed sequence. If a typed token did not match its corresponding target word then an error was assumed to be present. The mismatching tokens were then reversed to match the order in which they were typed/intended and skip characters were removed from the typed sequence. The first mismatched character in the tokens, moving from beginning to end was taken to be an error and this was included in our following analyses. The time interval between the preceding keypress and the erroneous keypress was also extracted.

**Figure 5:**
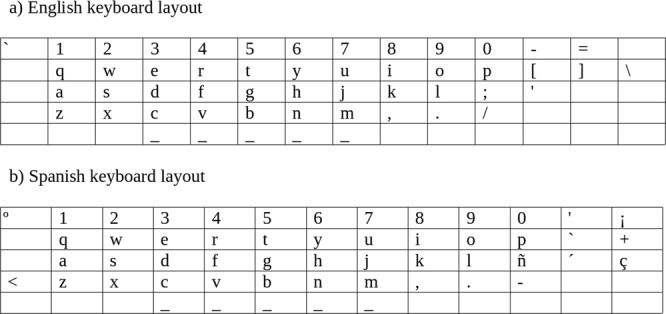
Keyboard layouts used by English and Spanish participants.

Once this error identification procedure had been performed, each error was categorised as a suspected motor error and/or a suspected habit error Motor errors were identified based on the keyboard distance from the preceding character to the typed character. If this distance was less than the distance from the preceding character to the target character then this was taken to be a potential motor error. Distance was calculated over the maps shown in Fig. 5, with identical coordinates used for secondary characters produced using the same key. The distance from a key to an adjacent key (including diagonally adjacent) was 1. The distance from that key to all other keys was the euclidean distance (using the depicted grid layout, Fig. 5. If a key is not found in the map (this includes ø, æ, *β*, Chinese, Greek, Cyrillic characters), then we calculate its distance as the euclidean distance from the preceding character to the average position on the keyboard. Capital characters or characters with diacritics are treated as “secondary characters”. For example, A is treated as a, and ç is treated as c.

Habit errors were identified by comparing the (back-off smoothed) conditional probability of the typed character given the preceding 4 characters and the (back-off smoothed) conditional probability of the target character given the preceding 4 characters. If p(typed|target-1…target-4) was higher than p(target|target-1…target-4) then the error was classified as a potential habit error. Assessed independently an error could be marked as both a motor and an habit error. For clarity of analysis and because habit errors are the focus on attention in this paper, only errors that were not motor errors were allowed to be habit errors.

Code for performing error identification and classification is publicly available at https://github.com/elisaF/typing-classification/.

## Data Availability

Full data on participants in not available, because consent was not obtained to share identifying and clinical information. However a sample of anonymised keypress information and analysis scripts which demonstrate the error identification and classification procedure are available at https://osf.io/2z8t9. The full code for error identification and classification are available at https://github.com/elisaF/typing-classification/. Scripts for gathering typing data are available at https://github.com/chbrown/typing-evaluation.
